# Personal Rights and the Criminal Law Amendment Bill

**Published:** 1917-05-26

**Authors:** 


### The Hospital

The Workers' Newspaper of Administrative Medicine and Institutional Life, Administration, National Insurance and Health.

No. 1616, Vol. LXII. SATURDAY, MAY 26, 1917. PRICE ONE PENNY

### PERSONAL RIGHTS AND THE CRIMINAL LAW AMENDMENT BILL.

With the motive and aims of the Criminal Law Amendment Bill now before Parliament it is hardly possible to imagine anything short of a general agreement. The diseases which the Bill is intended to check inflict upon many individuals a long account of bodily suffering, mental torture, and physical incapacity, while the economic loss they cause the nation reaches a serious total. Whatever can be done to lessen the tale of these disasters falls as a duty not less on statesmen than on humanitarians. The present Bill assumes that something can be secured by political regulations, and in particular by increasing the opportunities for expert treatment. To this central proposition of the Bill there does not seem to be any serious challenge. From it, however, there have emerged other issues, and some of these are exciting strong feeling among those who claim to stand strongly for the rights of personal freedom. One of these issues is raised by the clauses of the Bill which propose to inflict penalties upon persons, other than registered medical practitioners, who offer to undertake or actually do undertake the treatment of persons suffering from the diseases in question. In this particular sphere the intention is to suppress by legal enactment the existence of unqualified practice. Such a step, let it be freely admitted, is a new departure, and we can hardly wonder that it has encountered some hostile and even alarmed criticism. The break it makes with tradition offers one opportunity for assault, and the ease with which it is represented as the thin end of a wedge that is to be driven home at a later stage is a ready weapon for its opponents. "Here," it is said, "we have for the first time the compulsory driving of sick persons into medical consulting-rooms, and what is now intended in a limited sphere will soon be followed by wider restrictions; the rights of the free-born Briton are at stake, and a medical autocracy and monopoly intend to prevent the individual from taking advice in any direction he chooses, as is his present proud position." Inevitably, in such circumstances, there issue from certain quarters the usual screaming phrases about persecution, martyrdom, and the grasping and selfish tendencies of the medical profession; and the cry of "No tyranny" once more takes on a full sound.

With the rhetoric and rubbish of the agitators who cultivate a never-failing suspicion against any proposal which is based upon expert knowledge it would be idle to concern ourselves; and a plaintive protest from the professors of "medical herbalism . . . the hoariest of all the medical sciences," leaves us unmoved. But we are quite ready to recognise that in a number of persons who are honestly devoted to the cause of human freedom there is a certain fear lest this be invaded by the proposals now under discussion. If such persons exist, as we are convinced is the case, it is only fair to them that the position shall be stated in frank and open terms. This is the more necessary seeing that in the debate in the House one of the medical members of Parliament who dealt with the point here in question was not altogether happy in the manner in which he presented it. To judge from the brief report of the daily press, the speaker seemed to be hesitating between the free trade in medicine which exists to-day and a legal suppression of all unqualified practice. We believe him rightly to represent the majority of the profession when he spoke against the latter of these policies, and we are quite prepared to hold that to support the clauses of the present Bill is not inconsistent with this creed. Even if such a course were possible, we have no desire to inaugurate legislation intended to "put down" unqualified practice. By all means let every citizen be free to put the care of his health into the hands of any person he may think suited for so delicate a responsibility. So long as the matter concerns immediately and mainly his own comfort and welfare, we are fully disposed to let him take whatever course pleases him. But when the welfare, health, happiness and usefulness of other citizens are concerned, the State is surely entitled to take another and different note, and this both as a general protective duty and as a matter of cold economics. Now, this is the position of the diseases with which the Criminal Law Amendment Bill is concerned. Their effects are not limited to the individual patient. On the contrary, they may readily be communicated to others, and, more remotely, they may deprive the State of useful citizens by the effects they exercise on the reproduction of the race. Surely in such circumstances there is a public right or even a public duty to say that in the treatment of such diseases those who in all probability are competent shall alone be engaged. If this is granted, all that the Legislature can hope to do is to find a well-defined group of practitioners who have enjoyed a certain measure of training and experience and to commit to them the necessary work of treatment. Outside such a group there is no possibility of distinction between, say, the well-meaning enthusiast and the fraudulent impostor. Both alike are technically ignorant, and both are untrained. Hence, once allow that the treatment of venereal diseases concerns the public welfare, Parliament, apart from any suggestion of the medical profession, has no option but to decree that this treatment must be kept in the hands of those who possess the formal guarantee of a practical training and an examination diploma; and the motive for such a decree is not the establishment of a medical monopoly but the protection of the public interest.

